# Physical activity in urban green spaces: what do users value most in Casablanca, Morocco?

**DOI:** 10.1093/heapro/daag047

**Published:** 2026-04-09

**Authors:** Sammila Andrade Abdala, Celia Santos-Tapia, Filipe Canto Oliveira, Sara Arias, Safwane Mouwafaq, Asmae Khattabi

**Affiliations:** Mohammed VI International School of Public Health, Mohammed VI University of Sciences and Health, Boulevard Mohammed Taieb Naciri, Hay Hassani, Casablanca 82403, Morocco; Management and Public Health Laboratory, Mohammed VI Center for Research and Innovation, Boulevard Mohamed Al Jazouli, Madinat Al Irfane, Hay Riad, Rabat 10100, Morocco; Barcelona Institute for Global Health (ISGlobal), Carrer Rosselló 132, Barcelona 08036, Spain; Faculty of Health Sciences, International University of Casablanca, Route Provinciale 3020, Ville Verte, Bouskoura, Casablanca 50169, Morocco; Barcelona Institute for Global Health (ISGlobal), Carrer Rosselló 132, Barcelona 08036, Spain; Mohammed VI International School of Public Health, Mohammed VI University of Sciences and Health, Boulevard Mohammed Taieb Naciri, Hay Hassani, Casablanca 82403, Morocco; Management and Public Health Laboratory, Mohammed VI Center for Research and Innovation, Boulevard Mohamed Al Jazouli, Madinat Al Irfane, Hay Riad, Rabat 10100, Morocco; Mohammed VI International School of Public Health, Mohammed VI University of Sciences and Health, Boulevard Mohammed Taieb Naciri, Hay Hassani, Casablanca 82403, Morocco; Management and Public Health Laboratory, Mohammed VI Center for Research and Innovation, Boulevard Mohamed Al Jazouli, Madinat Al Irfane, Hay Riad, Rabat 10100, Morocco; National School of Public Health, Ministry of Health, Rue Lamfadel Cherkaoui, Madinat El Irfane, Rabat 10100, Morocco

**Keywords:** physical activity, urban green spaces, health promotion, urban health, participatory research

## Abstract

Urban green spaces (UGS) support physical activity and population health, yet their use depends on more than availability. In rapidly urbanizing cities, such as Casablanca (Morocco), understanding which UGS characteristics residents value is essential to inform equitable planning and health promotion. This study aimed to explore which characteristics of UGS users identify as motivating factors for engaging in physical activity and to analyze how these preferences vary across different sociodemographic groups in Casablanca. A cross-sectional survey was conducted in three UGS in Casablanca. A total of 468 participants completed structured questionnaires codeveloped through a participatory process under the Citizen Laboratory for Urban Health (CSU Lab). The survey assessed sociodemographic characteristics, physical activity behaviors, patterns of UGS use, and features valued for engaging in physical activity. Descriptive statistics, bivariate analyses, and multivariable logistic regression models were used. Proximity to home (69%), low pollution (43%), and safety (41%) were the top motivators. Functional aspects, such as facilities (36%), and environmental features, such as vegetation (33%), were also frequently cited. Preferences varied across population groups and contexts. After adjustment, proximity to home was more strongly valued by women, retired participants, and residents of specific districts, while younger adults were more likely to value facilities. Valuing safety was shaped primarily by place of residence, whereas valuing low pollution was less common among unemployed participants. This study demonstrates that urban planning and health promotion strategies must incorporate local user perspectives to effectively and equitably promote physical activity through UGS.

Contribution to Health Promotion

- Proximity to home is a key motivator for physical activity, particularly for women, retired participants, and residents of specific districts.
- Safety is an important motivator for physical activity, with safety concerns varying by place of residence.
- Facilities are more strongly valued by younger adults, indicating their relevance for strategies aimed at engaging this group in urban green spaces.
- To promote physical activity in urban green spaces, urban planning and health promotion strategies must address the preferences of different population groups to ensure equitable benefits.
- Participatory approaches are effective tools for supporting the implementation of health promotion strategies that reflect community needs.

## Introduction

As urbanization accelerates globally, cities—especially in low- and middle-income countries (LMICs)—face growing challenges in providing equitable access to quality urban green spaces (UGS). In many densely populated settings, limited availability of parks, gardens, and green corridors restricts opportunities for physical activity (PA), social interaction, and rest, especially among vulnerable groups (Shams and Barker 2019, Wang *et al*. 2019, Hunter *et al*. 2023).

UGS play a critical role in promoting health and well-being. They support regular PA, help reduce stress, improve cardiovascular and mental health outcomes, and offer important benefits for social cohesion (Akpinar 2016, Basu and Nagendra 2021). In contexts where free and accessible recreational settings are scarce, such as many LMICs, UGS therefore represent an essential health-promoting resource.

The mere presence of a UGS, however, does not guarantee its use. Several studies have emphasized that the quality, safety, accessibility, and amenities of UGS strongly influence whether people use them—especially for PA (Cohen *et al*. 2010, McCormack *et al*. 2010, Palliwoda and Priess 2021). In particular, vulnerable groups, including women, older adults, and low-income residents, may avoid UGS perceived as unsafe or poorly maintained, thereby exacerbating health inequalities (Basu and Nagendra 2021, Palliwoda and Priess 2021). Identifying the characteristics of UGS that enable or hinder use is critical for shaping supportive environments and guiding equity-focused urban planning and health promotion efforts.

Health behaviors, such as PA, are shaped not only by individual factors but also by interpersonal, community, environmental, and policy conditions, underscoring the importance of multilevel approaches (Sallis *et al*. 2015). To capture these complex influences, participatory approaches have been increasingly applied in health promotion, as they generate insights that are locally relevant and actionable while engaging communities in identifying solutions that reflect their needs and lived realities (Rubio *et al*. 2021, Mthembu *et al*. 2023, Nguyen *et al*. 2024). Such approaches also represent a departure from hierarchical research paradigms, fostering cocreated processes that can strengthen community capacity, enhance social impact, and improve policy relevance (Koster *et al*. 2012, Shirk *et al*. 2012). Recent studies have also demonstrated how participatory design of UGS can directly contribute to residents’ health (Oosterbroek *et al*. 2024). These developments resonate with the Ottawa Charter for Health Promotion (WHO 1986), which highlights supportive environments and community action as essential strategies for advancing health.

Nevertheless, existing research on what motivates PA in UGS has predominantly been conducted in high-income countries and with top-down observational or experimental methods, with few studies incorporating the perspectives of users—particularly in LMICs—into the design and evaluation of UGS (Ugolini *et al*. 2022). A recent systematic review (Abdala *et al*. 2025) identified only a few studies worldwide that used a participatory approach to address PA in UGS, most of which were researcher led, short term, and concentrated in high-income contexts, with limited inclusion of diverse community voices. This underscores the need for more equity-focused research in LMIC cities, such as Casablanca.

To our knowledge, this is the first study in Morocco to examine how users perceive and value UGS for PA. It is also the first in this context to actively involve citizen scientists in the research process, integrating their participation into both the design and implementation of the study. By applying a participatory framework through the Citizen Laboratory for Urban Health (CSU Lab) (Santos-Tapia *et al*. 2024), it contributes user-driven evidence to guide equitable urban planning and advance health promotion strategies in rapidly urbanizing LMIC contexts.

## Materials and methods

### Methodological framework

This study was guided by the CSU Lab (Santos-Tapia *et al*. 2024) framework—a participatory model developed in Barcelona by the Barcelona Institute for Global Health (ISGlobal) and LICHEN Social innovation, with support from the BIT HABITAT Foundation (Barcelona City Council). The framework was designed to promote community engagement in urban health research and innovation through a structured, bottom-up approach. Adapted for the first time in Casablanca, Morocco, the CSU Lab follows the four-phase structure proposed by Santos-Tapia *et al*. (2024): participatory diagnosis, citizen science, social innovation, and communication.

In this article, we report on the results of the citizen science phase, during which a community of citizen scientists—composed of university students and community members—decided to focus on the study of UGS as potential health assets, with the aim of understanding which specific characteristics promote their use for PA in the specific context of Casablanca. Together, participants codesigned the research protocol and selected user surveys and systematic park observations as methodological tools. The present study reports findings from the user surveys, while systematic observations and a full account of the CSU Lab process will be presented in separate publications.

### Study design and setting

This cross-sectional survey was conducted in three UGS in Casablanca, Morocco, and is reported in accordance with the STROBE (Strengthening the Reporting of Observational Studies in Epidemiology) guidelines (von Elm *et al*. 2007) (Supplementary File S1). Casablanca is Morocco’s largest city: the prefecture has about 3.2 million inhabitants, while the greater metropolitan area (Casablanca-Settat urban area) contains ∼5.6 million residents (Haut-Commissariat au Plan (HCP) 2024). Although Casablanca has seen recent municipal investments in park renovation and the development of new UGS, rapid urban expansion has reduced the proportion of UGS, and access to parks and recreational amenities remains uneven across neighborhoods (Derdouri *et al*. 2025). The three selected UGS included a large central park (Ligue Arabe Park), a smaller neighborhood park (Murdoch or ISESCO Park), and a recently developed greenway in front of the Mohammed VI University of Health Sciences (UM6SS). These sites were agreed upon in the CSU Lab meetings and selected to represent different types of UGS in the city, ensuring a diverse range of users.

### Data collection and participants

A nonprobabilistic, on-site intercept sampling strategy was used to recruit adult users of UGS in Casablanca. Participants were approached while using the selected sites during the data collection periods. As no sampling frame of UGS users was available, this approach enabled the recruitment of users present in real-life park settings. To promote heterogeneity of the sample, data collection was conducted across three UGS with contrasting characteristics, on both weekdays and weekends, and during morning and afternoon periods. Recruitment at each site continued until a similar number of questionnaires were completed, ensuring balanced representation across locations.

Data collection took place between late January and early February of 2025. Interviewers—citizen scientists supervised by the first author (S.A.A.)—were positioned at high-traffic areas within each site (e.g. main entrances and central pathways) and systematically approached individuals passing through these points to invite them to participate. As an incentive, participants were offered free health assessments, including body composition analysis using a bioimpedance scale and blood pressure measurement.

Eligible participants were adults aged 18 years or older who were present in one of the selected UGS, able to understand the questionnaire in Arabic or French (with assistance from citizen scientists if needed), and who provided informed consent. Individuals under 18 years of age or those who declined participation were excluded. Participation was voluntary, and all surveys were completed anonymously to ensure confidentiality.

### Questionnaire development and content validity

The questionnaire was codeveloped through a participatory process involving the research team and citizen scientists to ensure relevance to the local context and study objectives. Prior to full data collection, the instrument was tested within the participatory group both to assess clarity and comprehensibility of the items and to train citizen scientists in survey administration. This process contributed to establishing content validity (coverage and relevance of the domains) and face validity (clarity and apparent appropriateness of the questions). Minor wording adjustments were made after the initial field application to improve clarity. The full survey instrument is provided in Supplementary Files S2–S4.

Given the exploratory nature of the study and the fact that most variables were assessed using single-item indicators rather than multi-item scales, no formal psychometric reliability or construct validity testing was conducted.

### Variables

The questionnaire captured four main domains: (i) participant characteristics (sociodemographic information and self-reported chronic conditions); (ii) general PA indicators, including items adapted from the International Physical Activity Questionnaire (IPAQ) (Craig *et al*. 2003) short form; (iii) patterns of UGS use (e.g. frequency of park-based activity, timing and duration of visits, and typical activities undertaken); and (iv) perceived UGS characteristics and motivations related to engaging in PA.

The primary outcomes were motivations for engaging in PA in UGS, assessed through Question 15, which asked respondents to indicate which characteristics of UGS would encourage them to be more physically active. Multiple responses were allowed. Predefined options included proximity to home, accessibility, facilities, amenities, esthetics/attractions, safety, vegetation, fewer incivilities, and less pollution. Each motivator was treated as a binary outcome (selected vs not selected). Sociodemographic variables (sex, age, education, occupation, and district of residence) were included as explanatory variables. The other PA and park-use variables were used descriptively to characterize the sample but were not included in the bivariate and multivariable models reported in this article, which focused exclusively on motivations for PA in UGS.

### Data analysis

Survey responses were entered into Microsoft Excel and cleaned prior to analysis. Most variables had low levels of missing data. Higher nonresponse was observed for occupation (24.4%) and selected PA variables (15.6% for PA duration and 6.6% for PA duration in UGS), as well as for the chronic health conditions item (23.9%), which were not the focus of the present study.

Descriptive statistics were used to summarize participant characteristics and perceived motivators. Associations between sociodemographic variables and selected UGS characteristics were examined using chi-square tests and were conducted for the four most frequently reported motivators (proximity, low pollution, safety, and facilities) to focus on outcomes with sufficient prevalence and analytical relevance. Adjusted residuals were inspected in *post hoc* analyses to identify categories contributing most to statistically significant associations.

Multivariable logistic regression models were fitted separately for each outcome (proximity, low pollution, safety, and facilities) to identify factors independently associated with motivations for PA in UGS. A parsimonious modeling strategy was adopted to avoid overadjustment and preserve interpretability. Variables associated with each outcome at *P* ≤ 0.20 in bivariate analyses and/or considered conceptually relevant were eligible for inclusion in the multivariable models, resulting in separate final models for each outcome. Adjusted odds ratios (aORs) and 95% confidence intervals (95% CIs) are reported. Reference categories were defined *a priori* (female, age 18–24 years, employed, Ligue Arabe Park, and Hay Hassani district).

All analyses were conducted using Jamovi (version 2.6), with logistic regression models fitted in R via the Jamovi interface.

### Ethical approval

This study was approved by the Ethics Committee of the UM6SS in Casablanca. At the beginning of the questionnaire, a consent form explained the study’s purpose, voluntary nature of participation, and the possibility of publishing anonymized data. By completing the survey, participants indicated their informed consent.

## Results

### Sample characteristics

A total of 468 participants were surveyed across three UGS in Casablanca: Ligue Arabe Park (*n* = 159), Murdoch Park (*n* = 155), and the UM6SS greenway (*n* = 154). The sample was nearly evenly distributed by sex (49.9% female, 50.1% male). As shown in Table 1, the largest age group was the 50–64 (31.7%), while those aged 65 and over represented the smallest proportion (11.9%).

**Table 1:** Characteristics of the study sample, overall and by sex (*N* = 468).

Variable	Total (*N* = 468)	Female (*n* = 232)	Male (*n* = 233)
	*n* (%)	*n* (%)	*n* (%)
Recruitment location			
Ligue Arabe Park	159 (34.0%)	65 (28.0%)	91 (39.1%)
Murdoch Park	155 (33.1%)	73 (31.5%)	82 (35.2%)
UM6SS greenway	154 (32.9%)	94 (40.5%)	60 (25.8%)
Missing	0	0	0
Age group			
18–24	63 (13.9%)	31 (13.6%)	32 (14.2%)
25–34	92 (20.3%)	29 (12.7%)	63 (27.9%)
35–49	101 (22.2%)	65 (28.5%)	36 (15.9%)
50–64	144 (31.7%)	84 (36.8%)	60 (26.5%)
65+	54 (11.9%)	19 (8.3%)	35 (15.5%)
Missing	14	4	7
Education			
No formal education	49 (10.8%)	35 (15.8%)	14 (6.1%)
Primary/middle school	124 (27.3%)	70 (31.5%)	54 (23.5%)
High school/technical	153 (33.6%)	66 (29.7%)	86 (37.4%)
Undergraduate or more	129 (28.4%)	51 (23.0%)	76 (33.0%)
Missing	13	10	3
Occupation			
Employed	150 (42.4%)	46 (23.6%)	103 (65.6%)
Housewife	98 (27.7%)	98 (50.3%)	0 (0.0%)
Retired	45 (12.7%)	17 (8.7%)	28 (17.8%)
Student	32 (9.0%)	16 (8.2%)	15 (9.6%)
Unemployed	29 (8.2%)	18 (9.2%)	11 (7.0%)
Missing	114	37	76
Residence			
Hay Hassani	132 (29.7%)	77 (35.0%)	55 (24.9%)
Al Fida–Mers Sultan	99 (22.3%)	49 (22.3%)	50 (22.6%)
Casablanca Anfa	81 (18.2%)	39 (17.7%)	41 (18.6%)
Ain Sebaâ–Hay Mohammedi	41 (9.2%)	17 (7.7%)	24 (10.9%)
Ain Chock	32 (7.2%)	13 (5.9%)	19 (8.6%)
Ben M’Sick–Moulay Rachid	32 (7.2%)	11 (5.0%)	19 (8.6%)
Outside Casablanca	27 (6.1%)	14 (6.4%)	13 (5.9%)
Missing	24	12	12

*Note. N* = 468. Percentages are calculated based on valid responses per column. “Missing” rows reflect nonresponses for each variable. Because three participants did not report their gender, the sum of the stratified “Missing” counts may not always equal the “Total Sample” missing count. Categories for Residence are based on the eight official urban prefectures of Casablanca.

Stratification by gender revealed notable socioeconomic differences. Regarding education, while 28.4% of the total sample had completed undergraduate studies or higher, a larger proportion of the male column (33.0%) reached this level compared to the female column (23.0%). In contrast, women were more than twice as likely to report having no formal education (15.8% vs. 6.1% of men). The most pronounced disparity was observed in occupation: although 42.4% of the total sample was employed, this represented 65.6% of the male participants but only 23.6% of female participants. Furthermore, half of the female respondents (50.3%) identified as housewives. In terms of residence, the highest percentage of the total sample lived in Hay Hassani (29.7%), followed by Al Fida–Mers Sultan (22.3%) and Casablanca Anfa (18.2%).

### Descriptive overview of physical activity and park use

To contextualize the study population, general patterns of PA and park use were examined descriptively. About one-third of participants (35.2%) reported no vigorous PA in the past week. The most common durations were 30–90 minutes (47.6%) and less than 30 minutes (30.4%). Regarding PA duration within the UGS, the largest proportion of participants (43.4%) spent between 30 minutes and 1 hour engaging in activities, while 31.1% stayed for more than an hour, and 25.6% spent <30 minutes. Nearly half of the participants (49.0%) preferred practicing PA in UGS in the morning, while 20.9% preferred the afternoon and 17.1% the evening. Additionally, 12.8% marked more than one period of the day.

Regarding weekly usage of UGS, 35.9% of respondents used the UGS primarily during the weekend, 27.3% on weekdays, while 33.9% visited during both weekdays and weekends. Regarding the types of activities performed in the UGS, walking was the most prevalent activity, reported by 81.1% of participants, followed by relaxation (30.4%), running (21.7%), and enjoying the view (21.5%). Other activities were less frequent, including engaging in sports (11.5%), children’s play (10.2%), and cycling (5.2%).

### Urban green space characteristics that encourage physical activity

**Figure 1:**
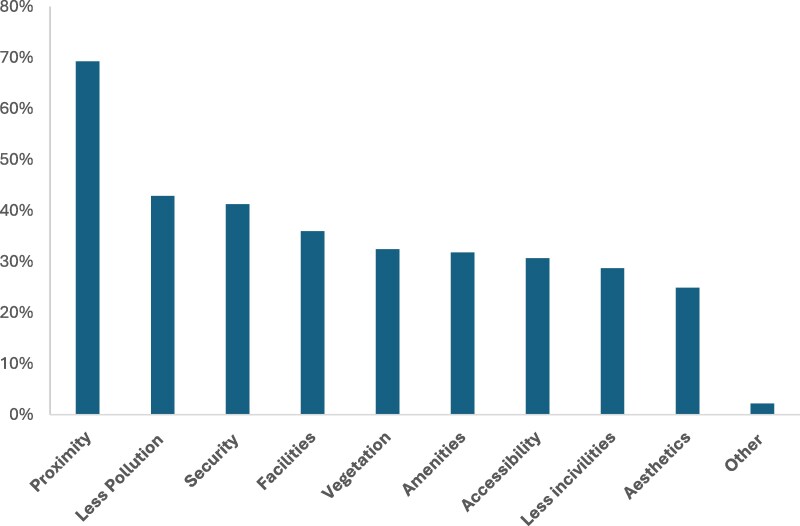
Urban green space characteristics valued for physical activity among survey respondents (*N* = 453).

When asked about the characteristics that motivated them to engage in PA in UGS, participants most frequently selected proximity to home (69.3%) (Fig. 1). This was followed by low levels of pollution (42.9%) and perceived security (41.3%). Functional aspects also played an important role: presence of facilities (36%) and general amenities (31.8%) were commonly reported as motivations for use. Vegetation (32.5%) and esthetic appeal (24.9%) reflected the importance of natural and pleasant surroundings, while accessibility (30.7%) and low levels of incivilities (28.7%) also influenced use. These findings indicate that both environmental conditions and infrastructure availability contribute to making UGS attractive for PA, and they emphasize the need to consider users’ preferences and local challenges in urban design and health promotion policies.

### Sociodemographic differences in valued urban green space characteristics for physical activity

Associations were examined using chi-square tests to assess relationships between participants’ sociodemographic profiles and the UGS characteristics they identified as important for motivating PA, with effect sizes ranging from small to moderate (Cramer’s *V* = 0.12–0.21) (Table 2).

**Table 2:** Chi-square analysis of associations between participant characteristics and reported motivators for physical activity in urban green spaces.

Variables	Chi-square	Cramer’s *V*	*P* value	Main residuals
Proximity × sex**	χ^2^(1) = 6.91	*V* = 0.124	0.009	Males slightly less likely to value proximity for PA (+1.49 no) and females more likely (−1.60 no)
Proximity × age group**	χ^2^(4) = 13.9	*V* = 0.178	0.008	Young adults (18–24) less likely to value proximity (+2.40 no, −1.82 yes)
Proximity × education	χ^2^(3) = 3.30	*V* = 0.086	0.347	No significant residuals
Proximity × occupation*	χ^2^(4) = 11.8	*V* = 0.185	0.019	Retired participants more likely to value proximity (−2.60 no, +1.43 yes)
Proximity × location	χ^2^(2) = 1.42	*V* = 0.056	0.493	No significant residuals
Proximity × residence	χ^2^(6) = 10.8	*V* = 0.159	0.095	No significant residuals; slight trend for the Ben M’Sick–Moulay Rachid group (+1.63 no)
Less pollution × sex	χ^2^(1) = 0.558	*V* = 0.352	0.455	No significant residuals
Less pollution × age group	χ^2^(4) = 3.37	*V* = 0.087	0.498	No significant residuals
Less pollution × education	χ^2^(3) = 7.35	*V* = 0.129	0.062	Not significant, but participants with higher education tended to value less pollution (−1.46 no, +1.58 yes)
Less pollution × occupation**	χ^2^(4) = 14.2	*V* = 0.204	0.007	Unemployed participants significantly less likely to value less pollution (+1.89 no, −2.61 yes)
Less pollution × location**	χ^2^(2) = 10.9	*V* = 0.155	0.004	Ligue Arabe users significantly more likely to value less pollution (+1.82 yes)
Less pollution × residence	χ^2^(6) = 9.40	*V* = 0.148	0.152	No significant residuals; slight trend for Ain Chock residents to value pollution more (+1.38 yes) and Al Fida less (−1.39 yes)
Security × sex	χ^2^(1) = 0.004	*V* = 0.002	0.949	No significant residuals
Security × age group	χ^2^(4) = 8.48	*V* = 0.139	0.075	No significant residuals, but adults 50–64 slightly less likely to value security (+1.32 no, −1.63 yes)
Security × education	χ^2^(3) = 1.38	*V* = 0.055	0.711	No significant residuals
Security × occupation*	χ^2^(4) = 10.5	*V* = 0.175	0.032	No significant residuals, but unemployed participants tended to value security less (+1.50 no, −1.91 yes), while students tended to value it more (−1.49 no, +1.43 yes)
Security × location*	χ^2^(2) = 8.86	*V* = 0.140	0.012	Murdoch users significantly less likely to value security (−1.67 yes)
Security × residence*	χ^2^(6) = 16.5	*V* = 0.196	0.011	Ain Chock (+1.52 yes) and Ain Sebaâ–Sidi Bernoussi (+1.42 yes) residents valued more security, while Al Fida–Mers Sultan residents were the least likely to prioritize it (−1.75 yes)
Facilities × sex	χ^2^(1) = 0.549	*V* = 0.034	0.459	No significant residuals
Facilities × age group**	χ^2^(4) = 18.9	*V* = 0.207	<0.001	Young adults (18–24: +1.70, 25–34: +1.86) tended to value facilities, while older adults (65+) tended not to (−1.91 yes)
Facilities × education	χ^2^(3) = 1.22	*V* = 0.052	0.747	No significant residuals
Facilities × occupation	χ^2^(4) = 4.71	*V* = 0.117	0.319	Not significant, but students tended to value facilities more (−1.08 no, +1.28 yes)
Facilities × location	χ^2^(2) = 2.87	*V* = 0.080	0.239	No significant residuals
Facilities × residence	χ^2^(6) = 3.76	*V* = 0.094	0.709	No significant residuals

Statistically significant associations are indicated as *P < 0.05 and **P < 0.01. Main residuals refer to adjusted standardized residuals; values exceeding |1.96| are considered statistically significant. Trends are noted for borderline results (0.05 < *P* < 0.10). *N* = 468 (total sample size; may vary slightly by variable due to missing data).

χ^2^, chi-square; *V*, Cramer’s *V*; PA, physical activity.

Proximity to home was significantly associated with sex, age group, and occupation. Men were less likely than women to identify proximity as a motivating factor [χ^2^(1) = 6.91; *P* = 0.009; *V* = 0.124]. Younger adults aged 18–24 years were also less likely to value proximity [χ^2^(4) = 13.9; *P* = 0.008; *V* = 0.178], whereas retired participants were more likely to prioritize it [χ^2^(4) = 11.8; *P* = 0.019; *V* = 0.185]. No significant associations were observed with education, park location, or district of residence, although a borderline trend was noted for residents of Ben M’Sick–Moulay Rachid.

Valuing low levels of pollution differed significantly by occupation and park location. Unemployed participants were significantly less likely to report low pollution as a motivating factor [χ^2^(4) = 14.2; *P* = 0.007; *V* = 0.204]. In contrast, users of Ligue Arabe Park were more likely to value low pollution compared with users of other sites [χ^2^(2) = 10.9; *P* = 0.004; *V* = 0.155]. Associations with education approached statistical significance, with participants of higher educational attainment tending to prioritize low pollution.

Perceived safety showed significant associations with occupation, park location, and district of residence. Unemployed participants tended to value safety less, whereas students tended to value it more [χ^2^(4) = 10.5; *P* = 0.032; *V* = 0.175]. Users of Murdoch Park were significantly less likely to identify safety as a motivating factor [χ^2^(2) = 8.86; *P* = 0.012; *V* = 0.140]. At the residential level, participants living in Ain Chock and Ain Sebaâ–Sidi Bernoussi were more likely to value safety, while those residing in Al Fida–Mers Sultan were the least likely to do so [χ^2^(6) = 16.5; *P* = 0.011; *V* = 0.196].

Valuing the presence of facilities was strongly associated with age group only. Younger adults (18–34 years) were more likely to identify facilities as motivating PA, whereas older adults aged 65 years and above were less likely to do so [χ^2^(4) = 18.9; *P* < 0.001; *V* = 0.207]. No significant associations were observed between facilities and sex, education, occupation, park location, or district of residence.

### Factors independently associated with key motivators for physical activity in urban green spaces

Multivariable logistic regression analyses identified several participant characteristics independently associated with key motivators for PA in UGS (Table 3).

**Table 3:** Multivariable logistic regression analysis of associations between participant characteristics and reported motivators for physical activity in urban green spaces.

Predictor	Proximity aOR (95% CI)	Less pollution aOR (95% CI)	Security aOR (95% CI)	Facilities aOR (95% CI)
Sex (ref: female)				
Male	0.47 (0.23–0.90)*	1.16 (0.66–2.04)	0.98 (0.54–1.76)	1.08 (0.72–1.63)
Age group (ref: 18–24)				
25–34	2.85 (0.95–8.83)	1.53 (0.52–4.69)	2.30 (0.75–7.32)	0.94 (0.48–1.80)
35–49	2.24 (0.75–6.82)	1.16 (0.40–3.50)	1.37 (0.45–4.26)	0.48 (0.25–0.93)*
50–64	2.58 (0.88–7.68)	1.19 (0.41–3.57)	0.62 (0.21–1.90)	0.44 (0.23–0.81)**
65+	1.27 (0.36–4.60)	1.02 (0.30–3.52)	0.77 (0.22–2.76)	0.27 (0.12–0.61)**
Occupation (ref: employed)				
Retired	4.98 (1.64–18.34)**	1.13 (0.50–2.53)	1.63 (0.70–3.78)	—
Student	1.12 (0.34–3.76)	1.12 (0.35–3.78)	2.05 (0.59–7.53)	—
Housewife	0.67 (0.29–1.49)	0.76 (0.38–1.52)	1.03 (0.50–2.13)	—
Unemployed	0.63 (0.25–1.66)	0.22 (0.06–0.64)**	0.41 (0.14–1.11)	—
Park location (ref: Ligue Arabe)				
Murdoch	—	0.37 (0.20–0.67)**	0.57 (0.27–1.21)	—
UM6SS	—	0.59 (0.32–1.06)	0.77 (0.35–1.74)	—
Residence (ref: Hay Hassani)				
Ben M’Sick–Moulay Rachid	0.20 (0.06–0.62)**	—	1.20 (0.33–4.32)	—
Ain Sebaâ–Hay Mohammedi	1.08 (0.40–3.08)	—	3.18 (1.17–9.08)*	—
Ain Chock	0.73 (0.27–2.03)	—	2.14 (0.73–6.58)	—
Outside Casablanca	0.68 (0.25–1.95)	—	1.09 (0.35–3.26)	—
Al Fida–Mers Sultan	0.98 (0.49–1.98)	—	0.98 (0.36–2.66)	—
Casablanca Anfa	1.15 (0.53–2.57)	—	1.64 (0.67–4.04)	—

Each outcome was analyzed using a separate parsimonious multivariable logistic regression model. Candidate variables were selected based on bivariate associations (*P* ≤ 0.20) and conceptual relevance. Education was excluded because of nonsignificance and high missingness (*n* = 144).

aOR, adjusted odds ratio; CI, confidence interval; —, variable not included in the final model.

Significance: *P < 0.05, **P < 0.01.

After adjustment, sex and occupation remained significantly associated with valuing proximity. Male participants were less likely than females to identify proximity as a motivating factor (aOR = 0.47; 95% CI, 0.23–0.90). Retired participants were substantially more likely to value proximity compared with employed participants (aOR = 4.98; 95% CI, 1.64–18.34). Residence was also independently associated with proximity, with participants living in Ben M’Sick–Moulay Rachid being less likely to prioritize proximity than those residing in Hay Hassani (aOR = 0.20; 95% CI, 0.06–0.62).

For low pollution, occupation and park location emerged as independent predictors. Unemployed participants were significantly less likely to value low pollution as a motivator for PA (aOR = 0.22; 95% CI, 0.06–0.64). Compared with users of Ligue Arabe Park, Murdoch Park users were also less likely to identify low pollution as motivating (aOR = 0.37; 95% CI, 0.20–0.67), while no significant differences were observed for UM6SS users.

Regarding safety, few variables remained significant after adjustment. Participants residing in Ain Sebaâ–Hay Mohammedi were more likely to prioritize safety compared with those living in Hay Hassani (aOR = 3.18; 95% CI, 1.17–9.08). No independent associations were observed for sex, age group, occupation, or park location.

Age group was the only factor independently associated with valuing facilities. Compared with young adults aged 18–24 years, participants aged 35–49, 50–64, and 65 years and older were significantly less likely to identify facilities as a motivating factor for PA, with the strongest inverse association observed among those aged 65 years and above (aOR = 0.27; 95% CI, 0.12–0.61).

## Discussion

This study explored which characteristics of UGS motivate users in Casablanca to engage in PA, with a focus on differences across sociodemographic groups. The most reported motivators were proximity to home (69%), low levels of pollution (43%), and perceived safety (41%). Bivariate analyses revealed that preferences for these features varied by age, sex, occupation, and residence. These findings underscore the need for inclusive urban planning that accounts for diverse user experiences and constraints.

Proximity to home was the most frequently cited motivator for PA in UGS. This aligns with other studies (Akpinar 2020), which showed that shorter walking distance to UGS significantly increased adolescents’ likelihood of visiting and being active, with distance and safety concerns being particularly important barriers for girls. A review of US studies found that people living closer to parks were sometimes more likely to be physically active, although findings were mixed. Stronger associations tended to appear when parks were nearby and perceived as safe and accessible (Bancroft *et al*. 2015). On the other hand, a Danish study found no significant link between actual distance to UGS and overall PA levels, indicating that features and quality of parks may play a more important role than proximity alone (Schipperijn *et al*. 2013). Evidence from New York City suggests that perceived accessibility to parks may play a stronger role than objective distance in shaping use and related health benefits, with social context—such as perceived safety—moderating this relationship (Orstad *et al*. 2020). Recent guidelines such as the 3–30–300 rule further stress the importance of proximity, recommending that all residents should live within 300 m of a public UGS to encourage regular use and associated health benefits (Konijnendijk 2023). In Casablanca, the high importance placed on proximity may reflect the scarcity of UGS and mobility limitations among groups, such as women and older adults.

Less pollution was the second most cited characteristic valued for PA in UGS. Numerous studies show that both perceived and objective air pollution can discourage outdoor PA. A systematic review and meta-analysis (An *et al*. 2018) found consistent evidence that poor air quality discourages PA. In particular, each small increase in fine particulate matter (PM2.5) was linked to higher odds of physical inactivity among adults. A subsequent review focusing in China showed that while PM2.5 reduced outdoor activity, perceptions of pollution often had an even stronger impact on behavior (An *et al*. 2019). Studies from China consistently show that increases in ambient PM2.5 significantly reduce moderate-to-vigorous PA among university students (Yu *et al*. 2017, 2021), and perceptions of pollution further discourage participation in outdoor or green exercise (Fan *et al*. 2021). Therefore, improving both actual air quality and its perception is critical to encouraging PA in UGS.

Security was cited by 41% of participants as a key factor influencing their PA in UGS. A large Norwegian study (Juul and Nordbø 2023) found a significant positive relationship between perceived neighborhood safety and PA levels, with each one-point increase in perceived safety corresponding to an increase of 6 minutes of weekly activity. Similarly, a study in the northeast of China (Bao *et al*. 2023) reported that perceptions of safety were among the most influential factors determining park visitation and PA in Chinese cities. In the context of European cities it was found that feelings of insecurity reduced park use, especially in socioeconomically disadvantaged neighborhoods (Fontán-Vela *et al*. 2021). This highlights the importance of designing UGS that not only reduce actual risks but also promote a sense of safety through visibility, maintenance, and inclusive use.

In our study, 36% of participants identified facilities—such as sports courts, walking paths, and playgrounds—as key motivators for PA in UGS. Evidence from the USA shows that adults with access to diverse or low-cost recreational facilities, such as parks, trails, and courts, are significantly more likely to use them for PA and to meet recommended activity guidelines (Kaczynski *et al*. 2014, Andrade *et al*. 2021). Together, these findings reinforce the role of built facilities in supporting active behaviors, particularly among younger and middle-aged adults.

### Key determinants of motivations for physical activity in urban green spaces

The results of this study indicated that preferences for key UGS features in promoting PA were shaped by a limited set of robust sociodemographic factors once confounding was taken into account. After adjustment, proximity to home remained significantly associated with sex and occupational status. Male participants were less likely to value proximity as a motivator for PA, while retired participants showed a markedly higher likelihood of prioritizing proximity. This contrasts with previous research suggesting that proximity is more strongly associated with walking among nonretired adults while playing a weaker role for retirees (Van Cauwenberg *et al*. 2015). The stronger emphasis placed on proximity by retired participants in our study may reflect contextual differences related to daily mobility patterns, reliance on the immediate neighborhood, or local urban conditions. Regarding gender-related differences, our findings are consistent with previous research showing that women are more likely than men to prefer opportunities for PA close to home (Van Uffelen *et al*. 2017), possibly due to safety concerns or household responsibilities. These results highlight the need to tailor UGS planning to accommodate different life stages and gendered patterns of park use.

In the adjusted analysis focusing on low levels of pollution as a motivating factor, unemployed participants were significantly less likely to prioritize this characteristic for engaging in PA in UGS, suggesting a link between employment status and environmental priorities. This supports findings in Bangladesh (Alam and Zakaria 2021), reporting that employment and income levels significantly predict environmental concern, with employed individuals showing greater awareness. Although education did not emerge as a significant determinant in our adjusted models, broader evidence suggests that educational attainment is often associated with environmental attitudes. For example, a large cross-national study found that education was a consistently strong predictor of proenvironmental preferences across 62 developed and developing countries, while the relationships with employment and income were more complex (Qadri *et al*. 2025).

Regarding safety, our adjusted analysis indicates that perceived security was primarily shaped by place of residence rather than individual sociodemographic characteristics. Participants living in Ain Sebaâ–Hay Mohammedi were more likely to prioritize safety as a motivating factor for engaging in PA in UGS, highlighting the role of neighborhood context in shaping safety concerns. This finding aligns with a broader body of literature emphasizing perceived safety as a key condition for promoting PA in UGS. Previous studies have shown that neighborhood greenness is associated with higher levels of PA only when individuals feel safe in their environment (Weimann *et al*. 2017) and that higher perceived safety itself is directly associated with increased weekly activity regardless of UGS availability (Juul and Nordbø 2023). Ensuring safe conditions within UGS is therefore essential to support equitable access and encourage their use for PA.

Finally, with respect to facilities, age emerged as the only determinant consistently associated with valuing this feature, a pattern that remained robust after adjustment. Younger adults were more likely to identify facilities in UGS as motivators for PA, whereas older adults were significantly less likely to do so. This age-based gradient aligns with previous research indicating that younger users tend to be more responsive to structured features that support active recreation. For instance, among adolescents, the presence of features, such as a skatepark, courts, walking paths, equipment lighting, toilets, and more than 25 trees, was found to nearly triple the likelihood of park use for PA (Edwards *et al*. 2015). In contrast, older adults often prioritize comfort, accessibility, and visual rest over active-use facilities, with shade trees, seating, and pedestrian circulation—rather than high-intensity sporting infrastructure—emerging as key elements for park attractiveness and use in aging populations (Veitch *et al*. 2022).

It is worth noting that much of the existing literature on UGS and PA comes from high-income countries, while evidence from low- and middle-income settings remains limited. By providing data from Morocco, this study contributes to addressing that imbalance and adds insight from a rapidly urbanizing LMIC context. Such perspectives are essential to ensure that global recommendations are grounded in diverse realities.

### Policy implications

The findings of this study have several implications for urban planning and public health policy. First, the strong emphasis on proximity highlights the need for more evenly distributed UGS across Casablanca, particularly in underserved neighborhoods. Urban development should prioritize walkable, accessible green areas to promote PA for all residents, with special attention to women and older adults whose daily mobility may be more locally constrained.

Second, the importance of safety and environmental quality underscores that UGS planning must address local conditions that shape whether parks are perceived as usable and supportive of PA. Place-based interventions—such as improved lighting, maintenance, visibility, and pollution mitigation around UGS—are essential to ensure that residents feel safe and motivated to use these environments for PA, particularly in socioeconomically diverse urban settings.

Third, the variation in preferences across different sociodemographic groups suggests that one-size-fits-all approaches are insufficient. Tailored interventions—such as investing in sports infrastructure for youth or enhancing comfort and accessibility for older adults—can improve inclusiveness and impact. Such differentiated approaches can help ensure that UGS remain inclusive and supportive of PA across different stages of life.

Finally, the participatory model applied in this study demonstrates how embedding citizen perspectives can strengthen equity-focused health promotion. Scaling up such approaches in rapidly urbanizing LMIC contexts can help ensure that future UGS policies and developments are responsive, empowering, and sustainable.

### Strengths and limitations

To our knowledge, this is the first study to examine UGS users’ perspectives on PA in Morocco, helping to fill an important gap in evidence from LMIC settings. A key strength of this study is its participatory design: the survey was codeveloped with citizen scientists and translated into Moroccan Arabic to ensure cultural and linguistic relevance, which enhanced inclusiveness and local validity. Combining context-specific items with validated measures from the IPAQ further strengthened methodological rigor. The inclusion of three contrasting types of UGS—two of the most well-known and frequently used parks in Casablanca and one newly developed greenway—allowed us to capture a diverse range of user profiles and experiences in a city where high-quality public UGS remain relatively scarce.

However, some limitations must be acknowledged. First, the use of a nonprobabilistic, on-site intercept sampling strategy may have introduced selection bias, as only individuals present in UGS at the time of data collection were eligible to participate. However, data collection across multiple sites, days, and time periods was intended to capture a heterogeneous sample of UGS users and reduce systematic overrepresentation of specific user groups. Second, only three sites were included; nevertheless, these sites were deliberately selected as some of the most prominent and contrasting UGS in the city, making this study a valuable first step toward building an evidence base in the Moroccan context. Third, some items (e.g. self-reported chronic conditions) had higher nonresponse, which limited their use in certain analyses. Finally, although most survey items were developed through a participatory process to ensure strong contextual relevance and face and content validity, they should be considered exploratory rather than fully standardized measures, and future studies could build on this work by formally evaluating their psychometric properties, including validity and reliability.

## Conclusion

This study provides valuable insights on the UGS characteristics that motivate PA among residents of Casablanca and how these preferences differ across sociodemographic groups. Proximity to home, low pollution, safety, and the availability of facilities were the most frequently cited motivators. Proximity was particularly important for women and retired participants, highlighting the role of life stage and locally constrained mobility. Preferences for facilities followed a clear age gradient, with younger adults placing greater value on sports and recreational infrastructure. In contrast, safety concerns were shaped primarily by place of residence, underscoring the importance of neighborhood context, while lower prioritization of environmental quality among unemployed participants points to the influence of socioeconomic vulnerability on environmental perceptions.

These findings highlight the need for inclusive, context-sensitive UGS design that addresses diverse user priorities and promotes equitable opportunities for PA. By embedding citizen input through a participatory approach, this study contributes to the limited but growing evidence from LMICs and provides actionable knowledge to guide health promotion and urban planning in rapidly urbanizing cities.

## Supplementary Material

daag047_Supplementary_Data

## Data Availability

The anonymized raw survey dataset is available from the corresponding author upon reasonable request.
