# Supplementary material for: Physical activity in urban green spaces: what do users value most in Casablanca, Morocco?
Source: Health Promot Int. 2026 Apr 9;41(2):daag047. doi: 10.1093/heapro/daag047 (PMC13070294; doi:10.1093/heapro/daag047)
Supplement: daag047_Supplementary_Data [file daag047_supplementary_data.zip › Supplementary File 2 English survey.pdf]

Welcome to our survey on urban green spaces and physical activity in Casablanca! Your involvement in this research study, undertaken within the framework of the Citizen Laboratory for Urban Health project, is entirely voluntary and requires less than 5 minutes to complete. By continuing the survey, you indicate your consent to participate and confirm that you are 18 years or older. Your responses will be anonymized and used for research and publication purposes. Your contribution is crucial in helping us understand how urban green spaces influence physical activity in Casablanca.
Thank you for supporting our efforts to make Casablanca a healthier and more active community!
If you have any questions about the study, please contact Sammila Andrade Abdala at: sandradeabdala@um6ss.ma

1. How old are you? __________
2. What is your sex? ☐ Male ☐ Female

3. What is the highest level of education you have completed?
☐ No formal education ☐ Primary education ☐ Lower secondary education (middle school) ☐ Upper secondary education (high school) ☐ Short-cycle tertiary education (technical/associate degree) ☐ Bachelor's degree ☐ Master's degree ☐ Doctorate

4. What is your current occupation? ___________________________________

5. In which district of Casablanca do you currently reside?
☐ Al Fida ☐ Anfa ☐ Ain Chock ☐ Ain Sebaa ☐ Ben M'sick ☐ Bernoussi
☐ Hay Hassani ☐ Hay Mohammadi ☐ Maarif ☐ Mers Sultan ☐ Moulay Rachid
☐ Roches Noires ☐ Sbata ☐ Sidi Belyout ☐ Sidi Moumen ☐ Sidi Othmane
☐ Other: ___________________________

6. Do you have any health problems?
☐ Diabetes ☐ High blood pressure ☐ Heart disease ☐ Asthma ☐ Arthritis
☐ Other (please specify: _________________) ☐ None

7. During the last 7 days, on how many days did you do vigorous physical activities like heavy lifting, digging, aerobics, or fast bicycling?
☐ 0 days
☐ 1 day
☐ 2 days
☐ 3 days
☐ 4 days
☐ 5 or more days

8. How much time did you usually spend doing vigorous physical activities on one of those days?
☐ Less than 30 minutes
☐ 30–90 minutes
☐ 90–150 minutes
☐ 150–300 minutes
☐ More than 300 minutes

9. What is the approximate distance from your home to the nearest urban park (park, greenway, forest, etc.)?
☐ Less than 500 m (0–5 min walk)
☐ 500 m–1 km (5–10 min walk)
☐ 1–2 km (10–20 min walk)
☐ 2–5 km (20–45 min walk)
☐ More than 5 km (45+ min walk)
☐ Not sure / I don't know

10. How often do you use the following urban green spaces/parks for physical activity?

	Never	Rarely	Sometimes	Often	Always
Ligue Arabe	☐	☐	☐	☐	☐
Hermitage	☐	☐	☐	☐	☐
Murdoch	☐	☐	☐	☐	☐
Other________	☐	☐	☐	☐	☐

11. For which activities do you use the urban green spaces/parks you visit?

☐ Walking ☐ Running ☐ Sports on fields ☐ Children's play
☐ Informal games ☐ Enjoying the scenery ☐ Walking the dog ☐ Social activities
☐ Relaxing ☐ Cycling ☐ Other: __________________

12. What is the average duration of your physical activity sessions in urban green spaces?

☐ <15 min ☐ 15–30 min ☐ 30–45 min ☐ 45 min–1 h ☐ 1–1.5 h ☐ 1.5–2 h ☐ >2 h

13. At what time of day do you usually practice physical activity in urban green spaces?

☐ Morning ☐ Afternoon ☐ Evening ☐ Night ☐ I don't use them

14. When during the week do you use urban green spaces/parks more?

☐ Weekdays ☐ Weekend (Saturday or Sunday) ☐ Both weekdays and weekends
☐ I don't use them

15. Which characteristics/aspects of urban green spaces/parks would motivate you to do more physical activity?

☐ Proximity (green spaces near my home)
☐ Better accessibility (access, paths, bike lanes, parking, signage, disabled facilities, etc.)
☐ Better facilities (playgrounds, sports fields, skateparks, outdoor gyms)
☐ Better amenities (benches, bins, fountains, toilets, shelters, shade, etc.)
☐ Better aesthetics and attractions (views, maintenance, vegetation, fountains, public art, attractions)
☐ Better safety (lighting, visibility, road safety, CCTV, etc.)
☐ Better vegetation cover (type, quantity, and quality of trees, shrubs, grass, soil, etc.)
☐ Fewer incivilities (trash, alcohol/drugs, prostitution, vandalism, odors, etc.)
☐ Less pollution (air and noise pollution)
☐ Other: _______________________

16. How often do you socially interact with other people in the urban green spaces/parks you visit?

☐ Never ☐ Rarely ☐ Sometimes ☐ Often ☐ Always

If you are interested in participating in this community project to promote physical activity in Casablanca's urban parks, please leave your email address for further contact:____

Weight ______ BMI ______ Body fat % ______ Muscle % ______
Resting metabolism ______ Visceral fat ______ Body age ______
