# Supplementary material for: Physical activity in urban green spaces: what do users value most in Casablanca, Morocco?
Source: Health Promot Int. 2026 Apr 9;41(2):daag047. doi: 10.1093/heapro/daag047 (PMC13070294; doi:10.1093/heapro/daag047)
Supplement: daag047_Supplementary_Data [file daag047_supplementary_data.zip › Supplementary File 3 French survey.pdf]

Bienvenue à notre enquête sur les espaces verts urbains et l'activité physique à Casablanca! Votre participation à cette étude de recherche, faisant partie du projet Laboratoire Citoyen de Santé Urbaine, est volontaire et prendra moins de 5 minutes à compléter. En poursuivant l'enquête, vous indiquez votre consentement à participer et confirmez que vous avez 18 ans ou plus. Vos réponses seront anonymisées et utilisées à des fins de recherche et de publication. Votre contribution est cruciale pour nous aider à comprendre comment les espaces verts urbains influencent l'activité physique à Casablanca. Merci de contribuer à nos efforts visant à rendre Casablanca une communauté plus saine et plus active! Si vous avez des questions sur l'étude, veuillez contacter Sammila Andrade Abdala par e-mail: sandradeabdala@um6ss.ma

1. Quel âge avez-vous?__________
2. Quel est votre sexe? ☐ **Homme** ☐ **Femme**

3. Quel est le plus haut niveau d'éducation que vous avez atteint?
☐ Pas d'éducation formelle ☐ Éducation primaire ☐ Éducation secondaire inférieure (collège)
☐ Éducation secondaire supérieure (lycée) ☐ Éducation tertiaire de courte durée (diplômes techniques ou associés) ☐ Licence ☐ Master ☐ Doctorat

4. Quelle est votre occupation actuelle? ___________________________

5. Dans quel quartier résidez-vous actuellement à Casablanca? (Arrondissement)
☐ Al Fida ☐ Anfa ☐ Ain Chock ☐ Ain Sebaa ☐ Ben M'sick ☐ Bernoussi
☐ Hay Hassani ☐ Hay Mohammadi ☐ Maarif ☐ Mers Sultan ☐ Moulay Rachid
☐ Roches Noires ☐ Sbata ☐ Sidi Belyout ☐ Sidi Moumen ☐ Sidi Othmane
☐ Autre: ___________________________
6. Avez-vous des problèmes de santé?
☐ Diabète ☐ Hypertension artérielle ☐ Maladie cardiaque ☐ Asthme ☐ Arthrite
☐ Autre (veuillez préciser: _________________) ☐ Aucun

7. Au cours des 7 derniers jours, pendant combien de jours avez-vous fait des activités physiques vigoureuses telles que des travaux lourds, du jardinage, de l'aérobic ou du vélo rapide?
☐ 0 jour dans la semaine
☐ 1 jour dans la semaine
☐ 2 jours dans la semaine
☐ 3 jours dans la semaine
☐ 4 jours dans la semaine
☐ 5 jours ou plus dans la semaine
8. Combien de temps avez-vous généralement passé à faire des activités physiques vigoureuses lors d'une de ces journées?
☐ Moins de 30 minutes
☐ 30–90 minutes
☐ 90–150 minutes
☐ 150–300 minutes
☐ Plus de 300 minutes
9. Quelle est la distance approximative entre votre domicile et le parc urbain le plus proche (parc, promenade verte, forêt, etc.)?
☐ Moins de 500 mètres (0–5 min de marche)
☐ 500 m–1 km (5–10 min de marche)
☐ 1–2 km (10–20 min de marche)
☐ 2–5 km (20–45 min de marche)
☐ Plus de 5 km (45+ min de marche)
☐ Pas sûr / Je ne sais pas

10. À quelle fréquence utilisez-vous les espaces verts urbains/parcs urbains suivants pour l'activité physique?

	**Jamais**	**Rarement**	**Parfois**	**Souvent**	**Toujours**
Ligue Arabe	☐	☐	☐	☐	☐
Hermitage	☐	☐	☐	☐	☐
Murdoch	☐	☐	☐	☐	☐
Autre ________	☐	☐	☐	☐	☐

11. Pour quelles activités utilisez-vous les espaces verts urbains/parcs que vous fréquentez?

☐ Marcher ☐ Courir ☐ Activités sportives sur les terrains ☐ Jeux pour enfants
☐ Jeux informels ☐ Apprécier le paysage ☐ Promenade avec le chien ☐ Activités sociales
☐ Se détendre ☐ Cyclisme ☐ Autre: ___________________

12. Quelle est la durée moyenne de vos sessions d'activité physique dans l'espace vert urbain?

☐ <15 min ☐ 15–30 min ☐ 30–45 min ☐ 45 min–1 h ☐ 1–1.5 h ☐ 1.5–2 h ☐ >2 h

13. À quelle heure de la journée pratiquez-vous généralement une activité physique dans l'espace vert urbain/parc urbain?

☐ Matin ☐ Après-midi ☐ Soir ☐ Nuit ☐ Je ne fréquente pas

14. À quelle période de la semaine fréquentez-vous davantage les espaces verts urbains/parcs?

☐ Jours de semaine ☐ Week-end (samedi ou dimanche) ☐ Les deux en semaine et le week-end ☐ Je ne fréquente pas

15. Quelles caractéristiques/aspects dans un espace vert/parc vous motiveraient à faire plus d'activité physique?

☐ Proximité (espaces verts près de chez moi)
☐ Meilleure accessibilité (accès, chemins, pistes cyclables, parkings, signalisation, installations pour personnes handicapées, etc.)
☐ Meilleures installations (aires de jeux, terrains de sport, skatepark, salle de gym en plein air)
☐ Meilleures commodités (bancs, poubelles, fontaines, toilettes, abris, ombrage, etc.)
☐ Meilleure esthétique et attractions (vues, entretien, végétation, fontaine, art public, attractions)
☐ Meilleure sécurité (éclairage, visibilité, sécurité routière, surveillance vidéo, etc.)
☐ Meilleure couverture végétale (type, quantité et qualité des arbres, arbustes, herbes, sol, etc.)
☐ Moins d'incivilités (déchets, alcool/drogues, prostitution, vandalisme, odeurs, etc.)
☐ Moins de pollution (pollution de l'air et bruit)
☐ Autre: ________________________

16. À quelle fréquence interagissez-vous socialement avec d'autres personnes dans les espaces verts urbains/parcs urbains que vous fréquentez?

☐ Jamais ☐ Rarement ☐ Parfois ☐ Souvent ☐ Toujours

Si vous êtes intéressé à participer à ce projet communautaire visant à promouvoir l'activité physique dans les parcs urbains de Casablanca, veuillez laisser votre adresse e-mail pour un contact ultérieur : ____

Poids____ IMC____ Pourcentage de graisse corporelle _____ Pourcentage de muscles ____
Métabolisme de repos ______ Graisse viscérale ______ Âge corporel ______
