# Supplementary material for: Physical activity in urban green spaces: what do users value most in Casablanca, Morocco?
Source: Health Promot Int. 2026 Apr 9;41(2):daag047. doi: 10.1093/heapro/daag047 (PMC13070294; doi:10.1093/heapro/daag047)
Supplement: daag047_Supplementary_Data [file daag047_supplementary_data.zip › Supplementary File 4 Arabic survey.pdf]

مرحبًا بكم في استطلاعنا حول المساحات الخضراء الحضرية والنشاط البدني في الدار البيضاء! مشاركتكم في هذه الدراسة البحثية، التي تشكل جزءًا من مشروع مختبر المواطن للصحة الحضرية، طوعية وستستغرق أقل من 5 دقائق لإكمالها. بمواصلة الاستطلاع، فإنكم تشيرون إلى موافقتكم على المشاركة وتؤكدون أن عمركم 18 عامًا أو أكثر. سيتم إخفاء هويتكم في الإجابات التي تقدمونها، وستُستخدم لأغراض البحث والنشر. مساهمتكم مهمة جدًا لمساعدتنا على فهم كيف تؤثر المساحات الخضراء الحضرية على النشاط البدني في الدار البيضاء. شكرًا لمساهمتكم في جهودنا لجعل الدار البيضاء مجتمعًا أكثر صحة ونشاطًا!

إذا كانت لديكم أي أسئلة حول الدراسة، يُرجى الاتصال بـ *Sammila Andrade Abdala* عبر البريد الإلكتروني *sandradeabdala@um6ss.ma*

1.ما هو عمرك ؟ ____________________________

2. ما هو جنسك؟ رجل☐ امرأة ☐

3.ما هو أعلى مستوى تعليمي حققته؟

☐لا يوجد تعليم رسمي
☐التعليم الابتدائي
☐التعليم الثانوي الأدنى (المدرسة المتوسطة)
☐التعليم الثانوي العالي التعليم
☐العالي إجازة
☐درجة البكالوريوس
☐ الماجستير
☐دكتوراه

4.ما هي وظيفتك الحالية ؟___________________________________

5.في أي حي تقيم حاليًا في الدار البيضاء؟ (المقاطعة)

☐المعاريف ☐حي المحمدي ☐حي الحسني ☐برنوصي ☐بن مسيك ☐عين السبع ☐عين شق ☐أنفا
☐مولاي رشيد ☐ سيدي مومن ☐ سيدي بليوط ☐ سباتة ☐ سيدي عثمان ☐الصخور السوداء
☐ مرس السلطان ☐ الفداء ☐آخر _________________________

6.هل لديك مشاكل صحية؟ (*حدد جميع الإجابات التي تنطبق*)

☐داء السكري
☐ارتفاع ضغط الدم
☐أمراض القلب
☐ الربو
☐التهاب المفاصل
☐ آخر (يرجى التحديد: ________________)
☐ لا شيء

7. خلال السبعة أيام الماضية، كم يوماً مارست النشاطات البدنية القوية مثل رفع الأثقال الثقيلة، أو الحفر، أو التمارين الرياضية، أو ركوب الدراجة السريعة؟

☐ 0 يوم في الاسبوع
☐ 1 يوم في الاسبوع
☐ 2 أيام في الأسبوع
☐ 3 أيام في الأسبوع
☐ 4 أيام في الأسبوع
☐ 5 أيام أو أكثر في الأسبوع

8. غالبًا، كم قضيت من الوقت في ممارسة النشاط البدني القوي في أحد تلك الأيام؟

☐< 30 min ☐30 - 90 min ☐90 - 150 min ☐150 - 300 min ☐> 300 min

9.ما هي المسافة بين منزلك والمساحة الخضراء القريبة من منزلك؟

☐ أقل من 500 متر / 0-5 دقائق مشي
☐ بين 500 متر و1 كيلومتر / 5-10 دقائق مشي
☐ بين 1 و2 كيلومتر / 10-20 دقيقة مشي
☐ بين 2 و5 كيلومترات / 20-45 دقيقة مشي
☐ أكثر من 5 كيلومترات / 45 دقيقة أو أكثر من المشي
☐لست متأكدًا / لا أعرف

10. كم مرة تستخدم المساحات الخضراء الحضرية أو الحدائق التالية لممارسة النشاط البدني؟

	دائمًا	غالبًا	أحيانًا	نادراً	أبدًا
حديقة جامعة الدول العربية	☐	☐	☐	☐	☐
رميتاج	☐	☐	☐	☐	☐
مر ضوخ	☐	☐	☐	☐	☐
آخر__________________	☐	☐	☐	☐	☐

11. ما هي الأنشطة التي تمارسها في المساحات الخضراء الحضرية أو الحدائق التي تزورها؟

☐المشي ☐الركض ☐الأنشطة الرياضية على الملاعب ☐ألعاب للأطفال ☐ألعاب غير رسمية
☐الاستمتاع بالمناظر الطبيعية ☐التجول مع الكلب ☐الأنشطة الاجتماعية ☐الاسترخاء
☐ركوب الدراجات ☐آخر __________

12. ما هي مدة النشاط البدني في المتوسط في هذه المساحة الخضراء الحضرية أو الحديقة الحضرية؟

☐< 15 min ☐15–30 min ☐30–45 min ☐45 min–1 h ☐1–1.5 h ☐1.5–2 h ☐> 2 h

13. ما هو الوقت الذي تمارس فيه عادةً النشاط البدني في المساحة الخضراء الحضرية أو الحديقة الحضرية؟

☐صباحاً ☐بعد الظهر ☐مساءً ☐ليلاً ☐لا أرتاد

14. أي وقت في الأسبوع تزور فيه غالبًا المساحات الخضراء الحضرية أو الحدائق الحضرية؟

☐من الإثنين إلى الجمعة ☐السبت أو الأحد ☐أي يوم من أيام الأسبوع ☐لا أقوم بزيارتها

15.ما هي الخصائص في المساحة الخضراء الحضرية التي تحفزك على ممارسة المزيد من النشاط البدني؟

☐ القرب / وجود المساحات الخضراء بالقرب من منزلي
☐ تجهيزات ،سهولة الوصول / الولوج، الممرات، مسارات الدراجات الهوائية، مواقف السيارات، الإشارات للأشخاص ذوي الاحتياجات الخاصة، إلخ
☐ تحسين المرافق / مناطق اللعب، ملاعب الرياضة، مضمار التزلج، صالة رياضية بالهواء الطلق، إلخ
☐ تحسين الوسائل الراحة / المقاعد، صناديق القمامة، النوافير، دورات المياه، مأوى، ظل، إلخ
☐ إلخ ،المرافق الترفيهية ،تحسين الجمالية والجاذبية / المناظر، الصيانة، النباتات، النوافير ، الفن العام
☐ إلخ ، تحسين الأمان / الإضاءة، الرؤية، السلامة المرورية، كاميرا المراقبة
☐ تحسين التغطية النباتية / نوع وكمية وجودة الأشجار، الشجيرات، العشب، التربة، إلخ
☐ التجاوزات أقل / نفايات، كحول / مخدرات، دعارة، تخريب، روائح، إلخ
☐ التلوث أقل / تلوث الهواء والضوضاء
☐ آخر ________________________

16. كم مرة تتفاعل اجتماعياً مع الآخرين في المساحات الخضراء الحضرية أو الحدائق الحضرية التي تزورها؟

☐أبدًا ☐نادرًا ☐أحيانًا ☐ غالبًا ☐ دائمًا

إذا كنت مهتماً بالمشاركة في هذا المشروع المجتمعي لتعزيز النشاط البدني في الحدائق الحضرية في الدار البيضاء ،يرجى ترك بريدك الإلكتروني للتواصل لاحقا . ______________________________

_____(Body Fat) نسبة الدهون في الجسم _____(BMI) مؤشر كتلة الجسم _____(Weight) الوزن
_____(Visceral fat) الدهون الحشوية _____(Metabolism) معدل الأيض في الراحة _____(Muscle) نسبة العضلات
_____(Body age) العمر الجسمي
